# Comparison of the Micro-Shear Bond Strength of Resin Cements to CAD/CAM Glass Ceramics with Various Surface Treatments

**DOI:** 10.3390/ma16072635

**Published:** 2023-03-26

**Authors:** Gaye Sağlam, Seda Cengiz, Ayşegül Köroğlu, Onur Şahin, Neslin Velioğlu

**Affiliations:** 1 Department of Prosthodontics, Faculty of Dentistry, Zonguldak Bülent Ecevit University, Zonguldak 67600, Turkey; 2 Department of Prosthodontics, DCT Clinic, Antalya 07000, Turkey; 3 Department of Prosthodontics, Navadent Oral and Dental Health Policlinic, Zonguldak 67000, Turkey

**Keywords:** CAD/CAM, glass ceramics, resin cement, shear bond strength, surface treatment

## Abstract

This study aimed to compare the effect of acid etching, sandblasting, or silica coating on the micro-shear bond strength of dual-cured resin cements to computer-aided design and computer-aided manufacturing (CAD/CAM) glass ceramic materials. Feldspathic, lithium disilicate, and zirconia-reinforced CAD/CAM ceramics were divided into four groups: control group (C), no surface treatment; hydrofluoric (HF) group, 5% HF acid-etched; sandblasting (SB) group, abraded with 50 µm aluminum oxide (Al_2_O_3_) particles; silica-coated (CJ) group, abraded with 30 µm silica-modified Al_2_O_3_ particles. Roughness values were obtained by using a profilometer. The cements were condensed on the surface-treated specimens and a micro-shear bond test was conducted. The ceramic material (*p* < 0.001) and surface treatment type (*p* < 0.001) significantly affected the micro-shear bond strength values. HF acid etching can be recommended for the surface pretreatment of feldspathic, lithium disilicate, and zirconia-reinforced CAD/CAM ceramics. Better bond strengths can be obtained with HF acid etching than with sandblasting and silica coating.

## 1. Introduction

In recent years, new ceramic materials with high durability and good aesthetics have been used in dentistry. Since these materials are difficult to process with conventional manufacturing techniques, computer-aided design and computer-aided manufacturing (CAD/CAM), which is an advanced manufacturing technology, have been introduced [1]. CAD/CAM systems use ceramic blocks, including feldspathic, leucite, lithium disilicate, or zirconia-reinforced lithium silicate glass ceramics, and zirconia [2,3]. The structural properties of ceramic materials determine their clinical application; thus, clinicians must have knowledge about the composition of ceramics and surface-conditioning protocols [3].

In CAD/CAM systems, feldspathic ceramic is used for aesthetic restorations, such as veneers or anterior single crowns, or inlay/onlay/endocrown restorations [4]. With the introduction of lithium disilicate ceramics, modifications have been made to the material in order to increase strength and extend the indication range [5]. Lithium disilicate ceramic that includes 70% lithium disilicate crystals after sintering, offers high fracture resistance and better aesthetic results due to its content and it is used for full crown restorations, inlays, or onlays [2,6]. Zirconia-reinforced lithium silicate ceramics, consisting of lithium disilicate crystals embedded in a glassy matrix, containing 8–12% zirconia, are a novel phase of glass ceramics [7]. Despite the higher glass content, it is claimed that the homogeneous distribution of zirconia in the glassy matrix improves the physical properties of the material and allows the material to be used for single-unit restorations, implant supported restorations, and table-tops [7,8].

An optimal bond strength between ceramic, luting cement, and dental tissue is necessary for ceramic restorations to be clinically effective over the long-term. Various surface treatments, including sandblasting, silica coating, and hydrofluoric (HF) acid, have been suggested for improving the adhesion between glass ceramic surfaces and luting cements, facilitating micromechanical and chemical retention [9,10]. To improve the surface energy before applying the silane agent and to increase the real surface area for mechanical locking with the resin cement, HF acid selectively etches the glassy phase of vitreous ceramic [11]. Sandblasting with aluminum oxide (Al_2_O_3_) particles has improved the effectiveness of the ceramic surface and increased the resin cement–ceramic bond strength [12]. In silica coating systems, the surface is air-abraded with silica-modified aluminum trioxide particles, which are firmly embedded on the ceramic surface by means of blasting pressure [13].

Dual-polymerized resin cements are classified based on the adhesion mechanism as follows: etch-and-rinse, self-etching, or self-adhesive. Three-step etch-and-rinse adhesives involve multiple steps, so the application procedures are time-consuming and error-prone. Self-adhesive resin cements were created to make the cementation technique of adhesive restorations easier because they do not require a pretreatment or bonding agent, so the clinical application time is short and eliminates technical errors through reduced steps or the use of fewer products in the cementation procedure [14].

The new generation of CAD/CAM block zirconia-reinforced lithium silicate ceramics is the most recently used silicate ceramic on the market and is distinguished from other glass ceramic CAD/CAM blocks by the zirconium crystals they contain (8–12%). Although the silica content of zirconia-reinforced lithium silicate glass ceramic (56–64%) is similar to that of feldspathic and lithium disilicate glass ceramics, the present study was conducted because the increased zirconium ratio to strengthen glass ceramic suggests that the sensitivity of zirconia-reinforced lithium silicate ceramics to surface treatments may vary compared to glass ceramics. Although the bond strength of zirconia-reinforced silicate ceramics after different surface treatments have been investigated in the literature, the reports are controversial [15,16,17,18,19,20,21]. Therefore, the present study aimed to compare the effect of HF acid etching, sandblasting, or silica coating on micro-shear bond strength (µSBS) between two types of resin cements and feldspathic, lithium disilicate, and zirconia-reinforced CAD/CAM glass ceramics, which are the most commonly used CAD/CAM blocks for indirect restorations. The null hypothesis was that no significant difference would be obtained in the bond strength obtained by cementation to the feldspathic ceramic, lithium disilicate, or zirconia-reinforced glass ceramic CAD/CAM materials when the three surface treatments were examined.

## 2. Materials and Methods

The materials used in the study are listed in Table 1. A total of 240 slices, with a dimension of 12 × 14 × 1.5 mm^3^, were obtained from three different glass ceramic CAD/CAM blocks under water cooling using a low-speed precision cutting device (Micracut 201, Metkon, Bursa, Turkey). The thickness of the samples was checked with a digital caliper (Alpha Tools, Mannheim, Germany). One surface of each specimen was flattened using silicon carbide abrasive papers (600, 800, 1000-grit) under water with a grinding machine (Gripo 2V, Metkon, Bursa, Turkey) for 15 s to achieve a standardized surface. The slices were divided into three groups according to the ceramic type: a feldspathic (VM), a lithium disilicate (EC), and a zirconia-reinforced lithium silicate (VS) (n = 80).

The EC and VS specimens were crystallized in a porcelain furnace (Programat EP 3000; Ivoclar Vivadent, Schaan, Liechtenstein) according to the manufacturer’s instructions. A polyvinyl chloride cylinder mold (16 mm × 12 mm) was filled with auto-polymerizing acrylic resin (PalapressVario, Heraeus Kulzer, Wehrheim, Germany), and next, the specimen was placed in the center of the mold with the flattened surface on top. Based on the surface treatment, all specimens of each type of ceramic material were randomly assigned to four groups (n = 20). In the control group (C), no surface treatment was completed. For the HF acid etching group (HF), the surfaces of the VM specimens were etched with 5% HF acid for 60 s, and the surfaces of the VS and EC specimens were etched with 5% HF acid for 20 s. Etching the surfaces of ceramics with HF acid was carried out according to the manufacturers’ instructions. In the sandblasted group (SB), the specimens were abraded with 50 µm Al_2_O_3_ particles for 10 s at a distance of 5 mm. The sandblasting process was carried out under 0.2 MPa pressure and the sandblasting nozzle (Bego sandblaster, Bego GMBH, Bremen, Germany) was placed perpendicularly to the ceramic surface. In the silica-coated group (CJ), the specimens were abraded with 30 µm silica-modified Al_2_O_3_ particles for 10 s, at a 5 mm distance from the surface. Abrading with the silica-coated particles was performed under 0.28 MPa pressure and the blasting nozzle (Cojet system, 3M ESPE, Seefeld, Germany) was held perpendicular to the ceramic surface. Then, the treated surfaces were ultrasonically cleaned with distilled water for 5 min and dried with air for 30 s. The surface roughness (Ra) of each specimen was measured three times using a contact profilometer (Surtronic 25, Taylor Hobson, Leicester, UK) with a tracing length of 4.0 mm and a cut-off length of 1.0 mm. The data were recorded, and mean Ra values calculated.

The specimens of control and each treated ceramic group were further assigned into two subgroups (n = 10) based on the type of cement: Variolink N/Monobond N (V) and BisCem/Porcelain Primer (B). Before placing the resin cements on the ceramic surfaces, all specimens were silanized by applying 1–2 thin coats of silane coupling agent (compatible with the resin cement system used) for 1 min and drying with air for 5 s.

The Variolink N base and catalyst (low viscosity, transparent shade) were mixed (1:1) for 10 s on a mixing pad and placed in a delivery syringe. A 2 mm high and 1 mm inner diameter polyethylene tube (Tygon S3 E-3603, Saint-Gobain, Courbevoie, France) was placed in the center of each ceramic surface, and the cement was condensed into the tube. The BisCem was condensed into the tube through an auto-mix dual-syringe and excess cement was removed with a transparent strip which was placed on the filled tube. Then the specimens were light-cured through the tube on each side for 10 s with an LED light-curing unit (Elipar S10, 3M Espe, St. Paul, MN, USA) at a light intensity of 1200 mW/cm^2^. Next, the tube was removed using scalpel blades. All specimens were stored in distilled water for 24 h at 37 °C prior to shear bond testing.

The specimens were attached to a shear bond tester (Bisco, Inc., Schaumburg, IL, USA) and the resin cement/ceramic surface interface was subjected to a force at a crosshead speed of 0.5 mm/min until failure. The bond strength of each specimen (MPa) was calculated by dividing the failure load (N) by the bonding area (mm^2^). A stereomicroscope (Leica EZ4 D, Leica Microsystems, Wetzlar, Germany) at ×10 magnification was used to obtain the failure pattern images, and the failure modes were classified as adhesive failure between the resin cement and the ceramic material, cohesive failure within the ceramic material, and mixed failure.

The statistical analysis was performed with a statistical software package (IBM SPSS v23, IBM Corporation, Armonk, NY, USA). Data were analyzed using the Shapiro–Wilk test to assess normal distribution, which showed that the data had normal distribution. Thus, three-way analysis of variance (ANOVA) was used to compare the micro-shearing values based on the type of material, surface treatment, and cement. Tukey’s honestly significant difference (HSD) test was used for multiple comparisons. Roughness values were analyzed using one-way ANOVA and Tukey’s test with a *p* of < 0.05. The chi-square test was used to compare the material and cement interaction groups based on the fracture types. *p* of <0.05 was considered to be statistically significant.

## 3. Results

**Figure 1:**
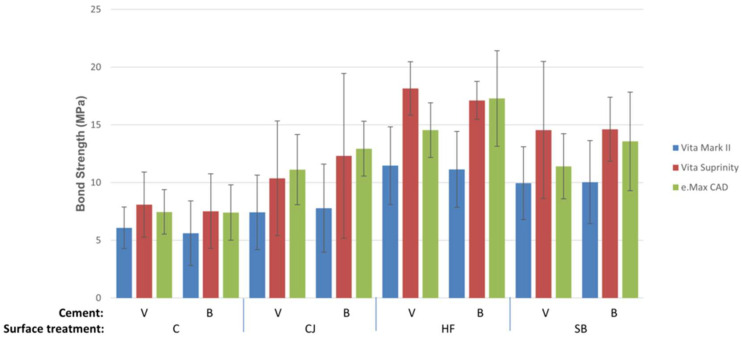
Mean and standard deviations of micro-shear bond strength values (MPa). C, control group; CJ, silica-coated group; HF, hydrofluoric acid etching group; SB, sandblasted group; V, Variolink; B, BisCem.

Means and standard deviations of the µSBS values (MPa) are shown in Figure 1. According to the three-way ANOVA analysis, the ceramic material and the surface treatment significantly influenced the bond strength values (*p* < 0.001) (Table 2). However, the type of resin cement did not have a statistically significant effect on the bond strength of the ceramic materials (*p* = 0.244). The two-factor interactions of surface treatment and ceramic materials were not significant (*p* = 0.119) and no statistically significant interaction was seen between the cement type and both ceramic material (*p* = 0.269) and the surface treatment (*p* = 0.636). The three-factor interaction between resin cement, surface treatment, and ceramic material was insignificant for the µSBS values (*p* = 0.929).

The results of the mean µSBS values of each tested ceramic showed that VS had the highest µSBS values (12.85 ± 5.50 MPa), followed by EC (11.97 ± 4.30 MPa); VM demonstrated significantly lower µSBS values (8.69 ± 3.70 MPa) in comparison to the other two ceramic materials (*p* < 0.001). In terms of surface treatment type, the highest total mean µSBS values were obtained in the HF group (14.96 ± 4.00 MPa), while the lowest values were seen in the control group (7.03 ± 2.58 MPa).

**Figure 2:**
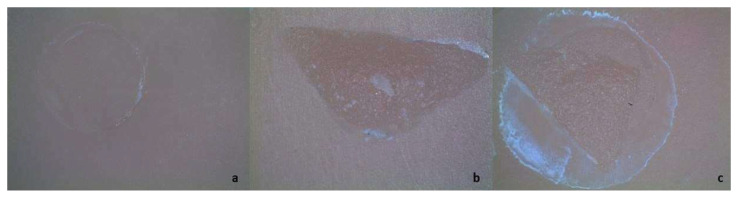
Stereomicroscopy photographs of the representative fracture patterns. Adhesive (**a**), cohesive (**b**), mix (**c**).

**Figure 3:**
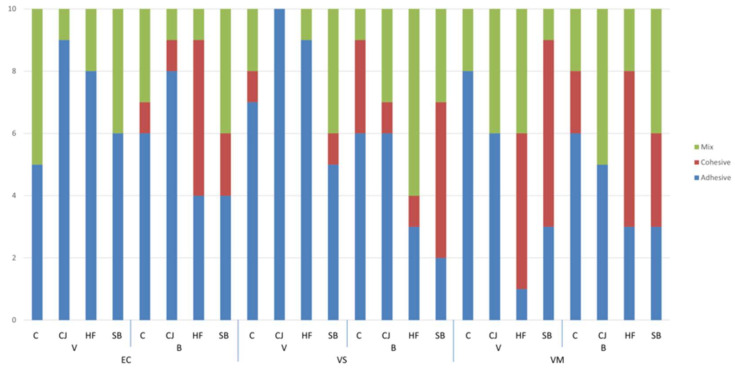
Fracture types of groups. VM, feldspathic; EC, lithium disilicate; VS, zirconia-reinforced lithium silicate; C, control group; CJ, silica-coated group; HF, hydrofluoric acid etching group; SB, sandblasted group; V, Variolink; B, BisCem.

Stereomicroscopy images determined the fracture patterns (Figure 2) and showed that the type of fracture was primarily adhesive failure between the cement and the ceramic material (55.4%), followed by mixed failure (27%), and cohesive failure (17.5%) (Figure 3). No significant difference was obtained between the distribution of the material and cement interaction groups according to fracture type in the C, CJ, and SB surface treatment groups (*p* values of 0.206, 0.101, and 0.072, respectively). The difference in the distribution of the cement interaction groups based on the kind of fracture was statistically significant in the HF group (HF acid surface treatment) (*p* < 0.001). This difference is due to the fact that the ratios of the VS-V, VS-B, and VM-V groups differed according to the fracture type.

Surface treatments affected the mean surface roughness values of glass ceramics (Table 3). The application of HF acid, sandblasting with Al_2_O_3_, and Cojet caused a statistically significant increase in the roughness of ceramic surfaces, when compared to control groups in each tested material (*p* < 0.05). Among the control groups of each ceramic, no significant difference was found (*p* > 0.05). The highest roughness values were obtained in the CJ and SB groups, without any statistically significant differences between the two groups, regardless of the type of ceramic.

## 4. Discussion

The adhesive interface of the bonded restorations consists of tooth-luting cement and luting cement restoration. Although studies have investigated the bond strength between teeth and cement [22,23], studies on the adhesion between ceramics and luting agents are needed due to the introduction of new ceramic restorative materials. The development of dental ceramics with different microstructure also influences the response to surface treatments applied to increase bond strength [24]. A durable bond strength between ceramic and luting agent can be achieved by mechanical and chemical retention provided by different surface treatments or a combination of them. Surface treatments such as acid etching or sandblasting provide micromechanical retention, whereas chemical retention is provided by silane application [25]. The present study aimed to analyze the µSBS between resin cements and ceramic materials with different surface treatments. The null hypothesis was partially rejected, because while the type of ceramic material or surface treatment affected the bond strength, the type of cement did not have an effect on bond strength.

In the present study, a µSBS test was used to assess the degree of bond strength between the ceramic material and resin cement. Micro-shear bond tests are applied to prevent unevenly distributed stresses that occur in conventional shear bond strength tests, and show more adhesive failures at the bonding interface than cohesive failures in the substrate, which is thought to be more realistic for evaluating the bonding ability of ceramic and resin luting cement [26].

Before cementing procedure with resin cements, different surface treatments are recommended for ceramics, such as mechanical roughing, silica coating, etching, sandblasting, or laser [9,27]. According to the present study findings, surface treatments had an impact on the bond strength to VM, EC, and VS materials and HF acid etching process presented higher bond strength values than the C, SB, and CJ groups in all ceramic materials (*p* < 0.001). It was stated that the etching mechanisms vary according to the type of abrasive, etching time, and ceramic microstructure and composition [28]. The glassy or crystalline phases of the restorative material are removed selectively by HF acid. The surface glassy state of the ceramic dissolves to a depth of several microns when it is etched with 5% to 10% HF, and pores appear on the surface. As a result, micromechanical retention is accomplished and the ceramic surface becomes rougher [11]. Acid etching enhances bond surface area and wettability by altering the surface energy of the ceramics; it also strengthens the resin bonding potential of the ceramic [29]. In this context, in the present study, 5% HF acid concentration was used for 20 s to roughen the surface of VS and EC, and for 60 s for VM, according to the manufacturer’s instruction.

The impact of etching protocols on the modification of the ceramic surfaces and the impact of these treatments on the binding strength to resin cement were previously assessed in a study and it is revealed that the most significant descriptive pore pattern was created by the HF (5–10%) treatment. Additionally, similar to the present study findings, it was shown that treatment with HF acid strengthened the shear bond between the ceramic material and the resin cement [30].

Although the combination of HF acid etching and a silane coupling agent is recommended as the gold standard for the surface conditioning of glass ceramics, it requires careful clinical use in order to protect the health of patients and physicians due to its caustic nature [15,31]. After the roughening of silica-based ceramics with hydrofluoric acid, by-products such as insoluble silica fluoride salts are formed on the surface, and if these are not removed, they can inhibit the bond strength of luting cement–ceramic by preventing the penetration of the resin cement into the microporous surfaces [32,33]. Sandblasting and silica coating can be used as alternative mechanical surface treatment methods to HF acid etching. Sandblasting positively affects the bond strength by increasing the ceramic wettability and surface area, but it is reported that it can cause microcracks that can lead to early failures on the ceramic surface [34]. In the present study, the bond strength of resin cement to sandblasted lithium disilicate ceramics was lower when compared to HF acid-etched ones. Similar to the results of the present study, Cınar et. al. reported that a surface treatment of lithium disilicate ceramics with sandblasting presented lower shear bond strength values than those treated with HF acid etching [35]. In addition, in the same study, they reported that more uniform micro-irregularities were obtained with HF acid application, while coarse surface irregularities were obtained with sandblasting for lithium disilicate ceramics in microscope images. The high shock energy of the Al_2_O_3_ particles during the silica coating process leads the silica particles to fuse to the ceramic surface, making the resin cement via the silane agent chemically reactive and improving the bond to ceramics [15]. In the literature, there are limited studies on silica coating of zirconia-reinforced lithium silicate glass ceramics. In the present study, CJ exhibited lower mean µSBS values (10.33 MPa) than HF (14.96 MPa) and SB (12.36 MPa) among all groups. The mean µSBS values of VS, according to the surface treatments, can be ranked as follows: HF (17.64 MPa) > SB (14.59 MPa) > CJ (11.35 MPa) > C (7.81 MPa) (*p* < 0.05). For zirconia-reinforced lithium silicates, previous studies have shown that HF acid etching was superior to sandblasting and silica coating for increasing µSBS values and sustaining long-term bond strength [15,24]. In accordance with the present study, Abdulkader et. al. [36] reported that HF (10%) acid etching for 20 s resulted in higher mean µSBS values in comparison to sandblasting with 50 μm aluminum oxide. However, some studies have shown that, regardless of the type of ceramic material, higher µSBS values were achieved with silica coating than HF acid etching [18,37]. This can be attributed to the differences in the test designs, the application method of the silica coating system, the type of composite resin, and the use of an adhesive resin.

The best surface preparation for cementing glass ceramics is HF acid etching followed by the application of a silane coupling agent [38]. The addition of silane to the etched surface improves wettability, forms a chemical adhesion between the methacrylate groups in the resin composite cement and the siloxane groups in silicate ceramic, and strengthens the adhesion between the two materials [39,40]. In the present study, silane was applied in accordance with the manufacturer’s instructions.

Samples without any surface treatment (control group) allowed us to see the effect of chemical bonding, since the ceramic surface is flattened with silicon carbide papers and has limited micromechanical retention compared to treated surfaces. Thus, the chemical bonding between the resin cement and the silanized surfaces was evaluated. When all the surface treatment procedures were compared, the significantly lowest bond strength values were obtained from control group (*p* < 0.001) for each ceramic material, when compared to HF, SB, and CJ, due to the cement’s limited ability to interlock. Although the ceramic surfaces had only been silanized, the failure modes at the ceramic surface/cement interface were primarily adhesive, indicating that ceramic surface pretreatment is necessary for silane to have an optimal bonding effect. These results are consistent with earlier research on glass ceramics [10,39,41].

According to the present study’s findings, the type of ceramic material affected the µSBS of the resin cements to the materials. When each type of ceramic material was compared, the VS specimens presented better bond strength values than the specimens with EC and VM (*p* < 0.001). Similar to the findings of a previous study [42], in the current study, the difference between VS and EC was not statistically significant for the mean bond strength values, independent of the conditioning protocol. Peumans et al. [16] compared the bond strength of two different types of resin cements to mechanical and chemical surface treatments applied to feldspathic, lithium disilicate, and zirconia-reinforced lithium silicate CAD/CAM materials. They concluded that the types of ceramic materials are affected differently by each surface treatment. It is seen that the application of silane and HF acid to glass ceramics significantly affects the bond strength. Acid application was found to be effective in zirconia-reinforced lithium disilicate blocks.

Among the ceramic materials investigated in the present study, HF acid treated zirconia-reinforced lithium silicate had better bond strength values. These findings are in accordance with the results reported by Sato et al., Ataol et al., and Altan et al., who stated that the highest bond strength values for Vita Suprinity was achieved with HF acid etching, when compared to sandblasting and silica coating [15,43,44]. The results obtained from the present study demonstrated that the bond strength values among the surface treatment types, including control groups, were lower for feldspathic ceramic (VM: 8.69 MPa) when compared to two other glass ceramics (VS: 12.85 MPa) (EC: 11.97 MPa). An in vitro study revealed lower shear bond strength values of resin cement for feldspathic ceramics compared to zirconia-reinforced lithium silicate ceramic and lithium disilicate ceramic [45]. Zirconia-reinforced lithium silicate consists of crystals of lithium metasilicate (Li_2_SO_3_), lithium orthophosphates (Li_3_PO_4_), and a glassy matrix with homogeneous dispersed zirconia particles (10 wt%). The crystal size in zirconia-reinforced lithium silicate ceramics (0.5–1.0 mm) is about 4–6 times smaller than in lithium disilicate ceramics (2.0–3.0 mm), which contributes to the higher glass content of zirconia-reinforced lithium silicate glass ceramics (50%) than lithium disilicate ceramics (about 30%) [21]. The unaffected areas after HF etching are lithium crystals in lithium-disilicate-reinforced ceramics, alumina crystals in feldspar ceramics, and lithium crystals and zirconium particles in zirconia-reinforced lithium silicate glass ceramics. Despite the similarities in silicate-containing glass ceramic structure, the high bonding values obtained in zirconia-reinforced lithium silicate ceramics can be attributed to the differences in microstructure of ceramics, amount of glass phase, crystal size, and the presence of a fine and uniform crystal structure exposed after etching with HF acid.

The present study revealed that self-adhesive resin cement or conventional resin cement has no effect on the bond strength of glass ceramics. Previous in vitro studies have stated that the bond strength between self-adhesive resin cements and composite or ceramic materials was lower in comparison to conventional resin luting cements [10,46,47]. Monomers with phosphoric acid groups are included in self-adhesive resin cements. In comparison to three-step standard resin cements, these agents are applied without the need for a separate adhesive system, and the clinical use of self-adhesive cements is straightforward, minimizing the possibility of handling errors and increasing their appeal to clinicians [48]. In the present study, the bond strength values were lower for the VM ceramic material than the EC and VS materials, regardless of the type of cement that was used. The lower bond strength might be due to the mechanical properties of the tested materials. VM is a silica-based CAD/CAM feldspathic ceramic with high biocompatibility, translucency, and aesthetic success. Feldspathic ceramics have lower fracture strength (130 MPa) than lithium disilicate (360 MPa) and zirconia-reinforced lithium silicate ceramics (370–420 MPa) [49,50]. The cohesive fracture within the ceramic material was 17.5% in this study and, among the cohesive failures, the highest rate was shown in VM. This suggests that the adhesion between the luting cement, the silane agent, and the ceramic structure is stronger than the internal strength of the feldspathic ceramic itself.

Surface roughness results obtained in this study showed that the surface roughness of all types of glass ceramics increased as a result of HF acid, sandblasting, and silica coating processes. The roughness values obtained with CJ and SB are higher than with HF. A previous study reported that no relationship could be established between surface roughness and bond strength in zirconia-reinforced lithium silicate ceramics [51]. In the present study, similar with the results in the literature [46,47,51,52,53], while the highest roughness values were obtained by sandblasting, the highest bond strength values were seen in the HF group for VS, and the other two ceramics. Sandblasting processes may cause crack formation and weakening of the ceramic structure by creating deep, irregular pits on the surface of glass ceramics, which do not present retentional features. [53,54]. When HF acid is applied, the glassy phase in glass ceramics dissolves and provides a uniformly microrough and porous surface. Another reason for obtaining these results may be the high elastic modulus of zirconium dioxide which forms microcracks that reduce the mechanical properties of the reinforced glass ceramics.

This in vitro study has some limitations. Although thermocycling has been shown to affect the luting cement–ceramic bonding due to changes in temperature and subsequent repeated contraction and expansion stresses [55,56], in the current study, initial bond strength was assessed without aging the specimens. In addition, further studies should be considered in clinical conditions to see the long-term success of bond strength between the resin cement and glass ceramics, as well as the surface treatment of both the tooth and the ceramic materials.

## 5. Conclusions

Within the limitations of this in vitro study, the following conclusions can be drawn:

1. Better bond strengths can be obtained with HF acid etching than with sandblasting and silica coating.
2. The application of silane alone may be considered insufficient to achieve adequate bond strength.
3. Self-adhesive or conventional resin cements can be used effectively in the cementation of glass-ceramic-based CAD/CAM restorations, and they are not superior to each other in terms of micro-shear bond strength values.
4. Prior to the resin cementation of feldspathic, lithium disilicate, and zirconia-reinforced CAD/CAM ceramic materials, HF acid etching can be recommended for surface pretreatment.

## Figures and Tables

**Table 1:** Materials used in the study.

Material and Type	Code	Composition	Manufacturer
Vitablocs Mark II; feldspathic ceramic	VM	SiO_2_ 56–64%, AI_2_O_3_ 20–23%, Na_2_O 6–9%, K_2_O 6–8%, CaO 0.3–0.6%, TiO_2_ 0.0–0.1%	Vita Zahnfabrik, Bad Säckingen, Germany
IPS e.max CAD; lithium disilicate glass ceramic	EC	SiO_2_ 58–80%, Li_2_O 11–19%, K_2_O 0–13%, ZrO_2_ 0–8%, Al_2_0_3_ 0–5%	Ivoclar Vivadent, Schaan, Liechtenstein
Vita Suprinity; zirconia-reinforced lithium silicate glass ceramic	VS	SiO_2_ 56–64%, ZrO_2_ 8–12%, Li_2_O 15–21%, Al_2_0_3_ 1–4%, K_2_O 1–4%, La_2_O_3_ 0.1%	Vita Zahnfabrik, Bad Säckingen, Germany
Variolink N; conventional resin cement	V	Bis-GMA, urethane dimethacrylate, triethylene glycol dimethacrylate, Ba-Al-fluorosilicate glass, barium glass, ytterbium trifluoride, spheroid mixed oxide, initiators, stabilizers, pigments	Ivoclar Vivadent, Schaan, Liechtenstein
Monobond N; silane coupling agent		Alcohol solution of silane methacrylate, phosphoric acid methacrylate, and sulfide methacrylate	Ivoclar Vivadent, Schaan, Liechtenstein
BisCem; self-adhesive, resin cement	B	Base: BisGMA, nonpolymerized dimethacrylate monomer, glass filler$Catalyst: Bis[2-(Methacryloyloxy)ethyl] Phosphate, 2-Hydroxyethyl Methacrylate, Bis(Glyceryl 1,3 Dimethacrylate) Phosphate, Dibenzoyl Peroxide	Bisco, Schaumburg, IL, USA
Porcelain Primer; silane coupling agent		Acetone, Ethanol, 3-(Trimethoxysilyl)propyl-2-Methyl-2-Propenoic Acid	Bisco, Schaumburg, IL, USA
IPS Ceramic Etching Gel	HF	5% hydrofluoric acid	Ivoclar Vivadent, Schaan, Liechtenstein
Korox 50	SB	50 mm Al_2_O_3_ particles	Bego GmbH, Bremen, Germany
CoJet Sand	CJ	30 mm silica-coated Al_2_O_3_ particles	3M ESPE, Seefeld, Germany

**Table 2:** Three-way ANOVA results of micro-shear bond strength values.

	df	MS	F	*p*
Material	2	345.527	27.723	<0.001
Surface treatment	3	604.418	48.495	<0.001
Cement	1	17.029	1.366	0.244
Material × Surface treatment	6	21.381	1.715	0.119
Material × Cement	2	16.493	1.323	0.269
Surface treatment × Cement	3	7.092	0.569	0.636
Material x Surface treatment × Cement	6	3.925	0.315	0.929

R^2^ = 0.533 (adjusted R^2^ = 0.477). df, degrees of freedom; MS, mean square; *p* < 0.05, significant difference.

**Table 3:** Means and standard deviations of surface roughness values for control and treated groups.

	n	C	CJ	HF	SB
VM	20	0.2772 ± 0.008 ^a^	1.043 ± 0.175 ^bc^	0.7161 ± 0.1225 ^c^	1.3716 ± 0.01 ^b^
EC	20	0.283 ± 0.062 ^a^	1.1674 ± 0.104 ^b^	0.82526 ± 0.2446 ^b^	1.1705 ± 0.3525 ^b^
VS	20	0.3001 ± 0.1 ^a^	1.2509 ± 0.03 ^b^	0.8048 ± 0.5215 ^c^	1.3255 ± 0.425 ^b^

Different lower-case letters in same row indicate statistically significant difference.

## Data Availability

The data presented in this study are available on request from the corresponding author.
